# Netupitant/palonosetron (NEPA) and dexamethasone for prevention of emesis in breast cancer patients receiving adjuvant anthracycline plus cyclophosphamide: a multi-cycle, phase II study

**DOI:** 10.1186/s12885-020-6707-9

**Published:** 2020-03-19

**Authors:** Roberta Caputo, Marina Elena Cazzaniga, Andrea Sbrana, Rosalba Torrisi, Ida Paris, Monica Giordano, Vincenzo Montesarchio, Valentina Guarneri, Laura Amaducci, Domenico Bilancia, Giuseppina Cilenti, Alessandra Fabi, Elena Collovà, Alessio Schirone, Erminio Bonizzoni, Luigi Celio, Sabino De Placido, Michelino De Laurentiis

**Affiliations:** 1 Breast Medical Oncology Division, Istituto Nazionale Tumori IRCCS “Fondazione G. Pascale”, Naples, Italy; 2 Medical Oncology Unit & Phase 1 Research Unit, ASST Monza, Monza, Italy; 3 Medical Oncology Unit, Department of Translational Research and New Technologies in Medicine and Surgery, University of Pisa, Pisa, Italy; 4 Department of Medical Oncology and Hematology, Humanitas Clinical and Research Center, Rozzano, Milan, Italy; 5 Division of Gynecologic Oncology, Fondazione Policlinico Universitario A. Gemelli IRCCS, Rome, Italy; 6 Medical Oncology Division, ASST-Lariana, Como, Italy; 7 Oncology Unit, “Monaldi” Hospital, Naples, Italy; 8 Department of Surgery, Oncology and Gastroenterology, University of Padova, Padova, Italy; 9 Istituto Oncologico Veneto IOV I.R.C.C.S, Padova, Italy; 10 Oncology Department Area Vasta Romagna, Faenza Hospital, Faenza, Ravenna, Italy; 11 Medical Oncology Unit, S. Carlo Hospital, Potenza, Italy; 12 Medical Oncology Division, Fondazione IRCCS Casa Sollievo Della Sofferenza, San Giovanni Rotondo, Foggia, Italy; 13 IRCCS Regina Elena National Cancer Institute, Rome, Italy; 14 Oncology Unit, ASST Ovest Milanese, Legnano Hospital, Legnano, Milan, Italy; 15 Clinical Oncology Division, Azienda Ospedaliero-Universitaria, Cona, Ferrara, Italy; 16 Department of Clinical Science and Community. Section of Medical Statistics, Biometry and Epidemiology “G.A. Maccacaro”. Faculty of Medicine and Surgery, University of Milan, Milan, Italy; 17 Medical Oncology Unit 1, Fondazione IRCCS “Istituto Nazionale dei Tumori”, Milan, Italy; 18 Clinical Medicine and Surgery Department, University of Naples Federico II, Naples, Italy

**Keywords:** NEPA, CINV, Nausea, Vomiting, Breast cancer, AC

## Abstract

**Background:**

NEPA is an oral fixed-dose combination of netupitant, a new highly selective neurokinin-1 receptor antagonist, and palonosetron. This study was conducted to evaluate whether the efficacy of NEPA against chemotherapy-induced nausea and vomiting (CINV) in cycle 1 would be maintained over subsequent chemotherapy cycles in breast cancer patients receiving adjuvant anthracycline plus cyclophosphamide (AC). The study also describes the relationship between efficacy on day 1 through 5 (overall period) and control of CINV on day 6 through 21 (very late period) in each cycle.

**Methods:**

In this multicentre, phase II study, patients received both NEPA and dexamethasone (12 mg intravenously) just before chemotherapy. The primary efficacy endpoint was overall complete response (CR; no emesis and no rescue medication use) in cycle 1. Sustained efficacy was evaluated during the subsequent cycles by calculating the rate of CR in cycles 2–4 and by assessing the probability of sustained CR over multiple cycles. The impact of both overall CR and risk factors for CINV on the control of very late events (vomiting and moderate-to-severe nausea) were also examined.

**Results:**

Of the 149 patients enrolled in the study, 139 were evaluable for a total of 552 cycles; 97.8% completed all 4 cycles. The proportion of patients with an overall CR was 70.5% (90% CI, 64.1 to 76.9) in cycle 1, and this was maintained in subsequent cycles. The cumulative percentage of patients with a sustained CR over 4 cycles was 53%. NEPA was well tolerated across cycles. In each cycle, patients with CR experienced a significantly better control of very late CINV events than those who experienced no CR. Among the patients with CR, the only predictor for increased likelihood of developing very late CINV was pre-chemotherapy (anticipatory) nausea (adjusted odds ratio = 0.65–0.50 for no CINV events on cycles 3 and 4).

**Conclusion:**

The high anti-emetic efficacy seen with the NEPA regimen in the first cycle was maintained over multiple cycles of adjuvant AC for breast cancer. Preliminary evidence also suggests that patients achieving a CR during the overall period gain high protection even against very late CINV events in each chemotherapy cycle.

**Trial registration:**

This trial was retrospectively registered at Clinicaltrials.gov identifier (NCT03862144) on 05/Mar/2019.

## Background

Cytotoxic chemotherapy remains an essential component for the management of breast cancer patients. Both gender and younger age affect the intrinsic emetogenicity of the chemotherapy regimen that still remains as the most important risk factor for chemotherapy-induced nausea and vomiting (CINV) [1]. Indeed, women have an increased risk of CINV, and younger patients (less than 50 years of age) are also more likely than older patients to develop CINV [1]. The combination of an anthracycline and cyclophosphamide (AC) is the backbone of the most effective adjuvant regimens for high-risk early-stage breast cancer. Although anthracyclines and cyclophosphamide are individually considered as being moderately emetogenic, it has been recognized that women receiving the combination of AC are at a particularly high risk of CINV [2]. Accordingly, the combination of AC is now classified as highly emetogenic chemotherapy (HEC) by international guidelines [3, 4]. The recommended anti-emetic prophylaxis consists of a triple regimen containing a 5-hydroxytryptamine-3 receptor antagonist (5-HT_3_RA), a neurokinin-1 receptor antagonist (NK-1RA), and single-dose dexamethasone. This regimen can help to control nausea and vomiting over the 5-day period of highest emetic risk after chemotherapy administration [4, 5]. It is also important to underline that successful CINV prevention in the first cycle of therapy should be sustained throughout all planned chemotherapy cycles [6].

Netupitant is a highly selective NK-1RA that exhibits a plasma half-life of approximately 96 h, making single-prophylaxis dosing appropriate [7]. Palonosetron is a pharmacologically distinct 5-HT_3_RA that demonstrates prolonged inhibition of 5-HT_3_ receptor function and inhibits 5-HT_3_-NK-1 receptor crosstalk [8, 9]. It should be noted that in breast cancer patients receiving AC anti-emetic guidelines updated from the Multinational Association of Supportive Care in Cancer recommend palonosetron as the preferred 5-HT_3_RA, when an NK-1RA is not available [4]. Synergy of netupitant with palonosetron has been demonstrated in vitro, suggesting the potential for an improved efficacy of this combination in clinical practice [8]. NEPA is an oral, single-dose, single-capsule, fixed-combination anti-emetic drug containing netupitant and palonosetron that is able to target the two major pathways involved in the transmission of emetic stimuli to the central nervous system during the acute and delayed phases of CINV [7]. In light of this, NEPA has the potential not only to simplify anti-emetic coverage but also improve guideline adherence in clinical practice with a convenient, single oral dose. The efficacy and safety of NEPA have been demonstrated in randomised trials involving chemotherapy-naive patients predominantly affected by solid tumors [7]. A pivotal trial in breast cancer patients treated with AC-containing chemotherapy demonstrated superior efficacy of a single dose of NEPA plus dexamethasone for CINV prevention, when compared with palonosetron plus single-dose dexamethasone in cycle 1 of therapy [10]. Studies have also shown NEPA to be well-tolerated over multiple cycles of emetogenic chemotherapy regimens [7]. It is important to highlight that a double-blind study in healthy subjects showed that administration of NEPA caused no significant effects on cardiac function, even at supra-therapeutic doses [11]. This is a very important issue especially in patients who are receiving chemotherapy containing potentially cardio-toxic agents such as the anthracyclines.

On the basis of this evidence, we decided to challenge NEPA efficacy and safety in a clinical setting where patients receive multiple cycles of the same chemotherapy regimen and anti-emetics should demonstrate a sustained benefit over all planned chemotherapy cycles. This phase II study was designed to evaluate whether the anti-emetic efficacy of NEPA plus single-dose dexamethasone observed in cycle 1 would be maintained over subsequent cycles of AC in patients with early-stage breast cancer. In addition, this study describes the relationship between anti-emetic efficacy in the overall study period (5 days after chemotherapy administration) and control of symptoms over the very late period (day 6 through 21 of a cycle) in a challenging setting of CINV.

## Methods

### Study design

This was a prospective, multicentre, single-arm, phase II study evaluating NEPA over four consecutive cycles of adjuvant chemotherapy including the combination of AC (doxorubicin or epirubicin plus cyclophosphamide) in breast cancer patients. The study was conducted in accordance with the Good Clinical Practice guidelines, at 22 Italian centers, which were coordinated by the GIM (Gruppo Italiano Mammella) cooperative group, from May to September 2016. Institutional ethics approval was granted at each participating center and written informed consent was obtained from each patient before enrolment.

### Patients

Female patients aged 18 years or over scheduled to receive AC-containing chemotherapy regimen for the adjuvant treatment of invasive breast carcinoma were eligible to participate. Patients were required to have an Eastern Cooperative Oncology Group (ECOG) performance status of 0, 1, or 2. Women of childbearing potential were also required to use reliable contraceptive measures during the study treatment. Patients had to be without episodes of emesis for 24 h before study entry, and no emesis because of any organic cause before study entry. Adequate hepatic and renal functions were required. Exclusion criteria included myocardial infarction within 6 months before study entry, uncontrolled diabetes mellitus, concurrent use of any drug with known anti-emetic efficacy, or presence of psychiatric or brain disorders that might interfere with ability to comply with study protocol.

### Interventions

The chemotherapy consisted of either doxorubicin intravenously (iv) (60 mg/m^2^) or epirubicin iv (90 mg/m^2^), each administered in combination with cyclophosphamide (600 mg/m^2^) iv on day 1 of a 21-day cycle. For all patients, anti-emetic coverage consisted of oral NEPA (netupitant 300 mg/palonosetron 0.50 mg) plus a single intravenous dose of dexamethasone 12 mg, both given before the administration of each chemotherapy cycle. NEPA was administered approximately 60 min before the start of chemotherapy on day 1. The use of rescue medications (metoclopramide and/or dexamethasone) for treatment of nausea and/or vomiting occurring within the 5 days after chemotherapy administration was considered treatment failure.

### Study outcomes

The primary efficacy end point of this study was CR (defined as no emesis, and no use of rescue medication) during the overall study period (day 1 through 5 post-chemotherapy). Secondary end points were the proportion of patients who achieved the following during the overall, acute (0–24 h post-chemotherapy), and delayed (day 2 through 5 post-chemotherapy) periods: CR (not including overall period), and no clinically significant nausea (defined as none to mild in severity). Nausea was graded daily using a four-point categorical Likert scale (0, none; 1, mild; 2, moderate; 3, severe), according to subjective assessment by each patient. All study patients were asked to complete a patient’s diary on a daily basis for the overall observation period (from the start of chemotherapy infusion on day 1 through the morning of day 6 of each cycle). Patients recorded daily any emetic episode and rescue medication intake as well as pre-chemotherapy experience (just before chemotherapy initiation) of either nausea or anxiety on a 11-point numerical rating scale (NRS; with 10 being the most severe) for each cycle. Pre-chemotherapy nausea and anxiety were defined a priori as a score of 1 or greater, and clinically significant pre-chemotherapy nausea and anxiety were defined as a score of 3 or greater [12]. At the end of the overall study period, each patient continued to complete the diary on a daily basis in order to capture any episodes of vomiting and/or nausea during the very late observation period (day 6 through 21) for each chemotherapy cycle. An exploratory efficacy end point was the proportion of patients without CINV events (vomiting and moderate-to-severe nausea) during the very late observation period in each cycle. Safety was assessed by treatment-emergent adverse events (TEAEs). Any serious TEAE judged by the investigator to be possibly, probably, or definitely related to the study treatment was recorded and graded according to the common terminology criteria for adverse events (CTCAE), version 4.3.

### Statistical analysis

The primary aim of this study was to evaluate the anti-emetic efficacy of NEPA plus single-dose dexamethasone based on the proportion of patients with a CR during the overall period of each cycle of AC-containing chemotherapy. The study was planned according to a one-stage Fleming design with a total sample size of 135 evaluable patients to decide whether the proportion of CR was > 64% (maximum response proportion of a poor anti-emetic regimen) during the overall period, with a type I error of 5% (one-sided) and type II error of 20% assuming a minimum response proportion of a good anti-emetic regimen equal to 74%. Considering an attrition of up to 10%, 150 patients were needed at baseline. In this study, the efficacy hypothesis was verified by resorting to two-sided confidence intervals (CIs) with 90% coverage, that were calculated using the Wald method. Therefore, the null hypothesis was rejected if the lower boundary of the 90% CI of the proportion of responders was greater than 64%. In spite of greater emphasis was on efficacy analysis in cycle 1, the study findings had to be confirmed also in the treatment cycles 2, 3, and 4. The efficacy analysis population was defined as all patients who received protocol required AC, study treatment and completed the patient’s diary in cycle 1. The safety analysis population consisted of all patients who received chemotherapy and study treatment.

An analysis of sustained overall (days 1 to 5) CR evaluated the probability that a patient would remain a responder over the subsequent cycles of chemotherapy. To accomplish this, a Kaplan-Meier curve was computed with patients who did not sustain a response, considered as treatment failures [13]. In post hoc analyses, we examined the association between overall CR and very late CINV events within each cycle, with comparison made using Fisher’s exact test. We also performed an exploratory analysis to examine the impact of risk factors for CINV such as age, motion sickness, pregnancy-related morning sickness, alcohol intake, and the pre-chemotherapy nausea and anxiety NRS at each cycle on very late CINV using logistic regression models. The analysis was restricted to patients who experienced a CR, and analyses were repeated for each cycle. All analyses were conducted using SAS version 9.4 (SAS Institute, Cary, NC, USA).

## Results

A total of 149 consecutive patients were enrolled in the study, and represent the safety population. Ten patients were excluded from the efficacy analysis (*n* = 3 patients withdrew the consent before starting the study, and *n* = 7 patients were not evaluable for anti-emetic efficacy), leaving 139 patients in the efficacy population. In this population, a total of 552 chemotherapy cycles were administered, and 97.8% of the patients completed the four planned cycles.

Baseline patient characteristics including emetic risk factors are shown in Table 1. The median age was 48 years, and the vast majority of the patients (94%) were treated with the double chemotherapy regimen containing an anthracycline (27% doxorubicin and 67% epirubicin) and cyclophosphamide.

**Table 1:** Patient’s demographic and clinical characteristics (enrolled population, *n* = 149)

Characteristic		NEPA plus 1-day Dex No. (%)
Age (years)		
Median	48	
min-max	25–76	
Age < 50 years		82 (55)
Height (cm)		
Mean	162.2	
SD	6.4	
Weight (kg)		
Mean	66.9	
SD	14.5	
ECOG performance status		
0		146 (98)
1		3 (2.0)
Chemotherapy regimen		
AC		40 (26.8)
EC		100 (67.1)
FEC		5 (3.4)
Other/missing		4 (2.7)
Alcohol consumption		
No		88 (59.1)
Occasionally^a^		42 (28.2)
Regularly		16 (10.7)
Missing		3 (2.0)
History of motion sickness		47 (31.5)
History of pregnancy-related morning sickness		57 (38.3)

*NEPA* fixed-dose combination of netupitant and palonosetron, *Dex* dexamethasone, *SD* standard deviationm, *AC* anthracycline (i.e., doxorubicin) and cyclophosphamide, *EC* epirubicin and cyclophosphamide, *FEC* fluorouracil, epirubicin, and cyclophosphamide

^a^It is defined as drinking one or two glasses per week

### Efficacy during the acute, delayed, and overall study periods

**Fig. 1:**
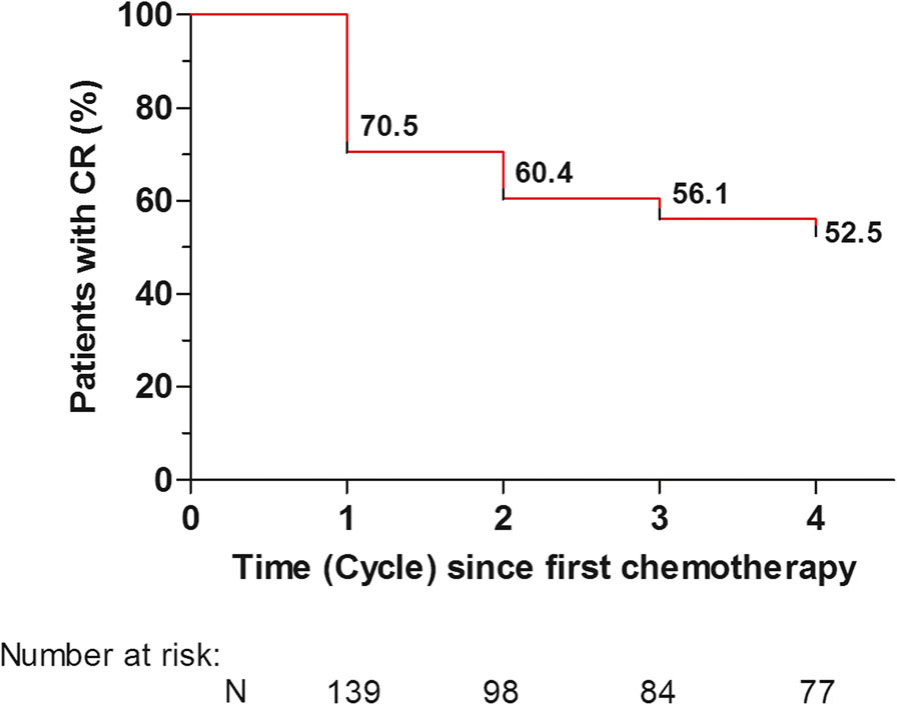
Kaplan-Meier curve of continued CR success rate. Patients who did not sustain a CR across cycles 1–4 were considered treatment failures. CR, complete response (no emesis, and no use of rescue medication)

The CR rates during the acute, delayed, and overall study periods are shown in Table 2. The proportion of patients with an overall CR was 70.5% (90% CI, 64.1 to 76.9) in cycle 1, and this was maintained in subsequent cycles. In each cycle, the primary end point was met because the lower limit of 90% CI always exceeded the preset cut-off of 64%. CR rates were similar across chemotherapy cycles during the acute and delayed periods. The percentage of patients who experienced a CR in cycle 1 and who sustained a CR over cycles 2–4 is shown in Fig. 1. The Kaplan-Meier curve showed that more than 50% of patients sustained a CR over cycles 2–4. No significant nausea rates were similar across cycles during the delayed and overall periods (Table 2).

**Table 2:** Efficacy end-point analysis (efficacy set population) in patients receiving a single dose of NEPA and dexamethasone

	Overall period (days 1 to 5)		Acute period (day 1)		Delayed period (days 2 to 5)	
	N (%)	90% CI	N (%)	90% CI	N (%)	90% CI
Cycle 1 (*N* = 139)						
CR	98 (70.5) a	64.1; 76.9^b^	119 (85.6)	80.7; 90.5	101 (72.7)	66.4; 78.9
NSN	80 (57.6)	50.7; 64.5	113 (81.3)	75.9; 86.7	85 (61.2)	54.4; 68.0
Cycle 2 (*N* = 139)						
CR	98 (70.5)	64.1; 76.9	118 (84.9)	79.9; 89.9	102 (73.4)	67.2; 79.6
NSN	79 (56.8)	49.9; 63.7	101 (72.7)	66.4; 78.9	81 (58.3)	51.4; 65.2
Cycle 3 (*N* = 138)						
CR	100 (72.5)	66.2; 78.7	115 (83.3)	78.1; 88.6	104 (75.4)	69.3; 81.4
NSN	85 (61.6)	54.8; 68.4	102 (73.9)	67.8; 80.1	88 (63.8)	57.0; 70.5
Cycle 4 (*N* = 136)						
CR	96 (70.6)	64.2; 77.0	106 (77.9)	72.1; 83.8	100 (73.5)	67.3; 79.8
NSN	82 (60.3)	53.4; 67.2	95 (69.9)	63.4; 76.3	87 (64.0)	57.2; 70.7

*NEPA* netupitant plus palonosetron, *CI* confidence interval, *CR* complete response (no vomiting and no use of rescue medication), *NSN* no clinically significant nausea (none to mild in severity)

aPrimary efficacy end point

^b^Efficacy hypothesis was demonstrated as the lower boundary of the 90% CI was greater than the preset cut-off of 64% which was assumed as the maximum response rate for a poor anti-emetic treatment in the study protocol

### Incidence of pre-chemotherapy nausea and anxiety

The incidence of pre-chemotherapy (anticipatory) nausea (score of ≥1) increased overall from cycle 1 to cycle 4, while the incidence of significant pre-chemotherapy nausea (score of ≥3) increased only from cycle 1 to cycle 3 (Table 3). The intensity of pre-chemotherapy nausea increased over each subsequent cycle, with intensity being almost doubled by cycle 3.

**Table 3:** Descriptive summary of pre-chemotherapy nausea and anxiety by chemotherapy cycle

	Cycle 1 (***n*** = 139)		Cycle 2 (***n*** = 139)		Cycle 3 (***n*** = 138)		Cycle 4 (***n*** = 136)	
Mean score for prechemotherapy nausea, (0–10 NRS)	0.85	0.56; 1.13	1.47	1.08; 1.85	1.64	1.26; 2.01	1.65	1.27; 2.02
Pre-chemotherapy nausea (≥1 NRS)	29 (20.9%)	15.8; 27.1	48 (34.5%)	28.2; 41.4	51 (36.9%)	30.5; 43.9	55 (40.4%)	33.8; 47.5
Significant prechemotherapy nausea (≥3 NRS)	19 (13.7%)	9.5; 19.2	25 (17.9%)	13.2; 23.9	37 (26.8%)	21.1; 33.4	34 (25%)	19.4; 31.6
Mean score for prechemotherapy anxiety, (0–10 NRS)	2.68	2.25; 3.10	1.81	1.46; 2.16	1.97	1.62; 2.33	2.03	1.66; 2.40
Pre-chemotherapy anxiety (≥1 NRS)	85 (61.2%)	54.2; 67.7	66 (47.5%)	40.6; 54.4	74 (53.6%)	46.6; 60.5	72 (52.9%)	45.9; 59.9
Significant prechemotherapy anxiety (≥3 NRS)	58 (41.7%)	35.1; 48.7	40 (28.8%)	22.9; 35.5	43 (31.2%)	25.1; 37.9	48 (35.3%)	28.9; 42.3

*CI* confidence interval, *NRS* numerical rating scale (with 10 being the most severe)

Data are reported with 90% CI

The incidence of pre-chemotherapy anxiety and significant pre-chemotherapy anxiety decreased across cycles (Table 3). Likewise, the intensity of pre-chemotherapy anxiety decreased across cycles.

### Efficacy during the very late period

**Fig. 2:**
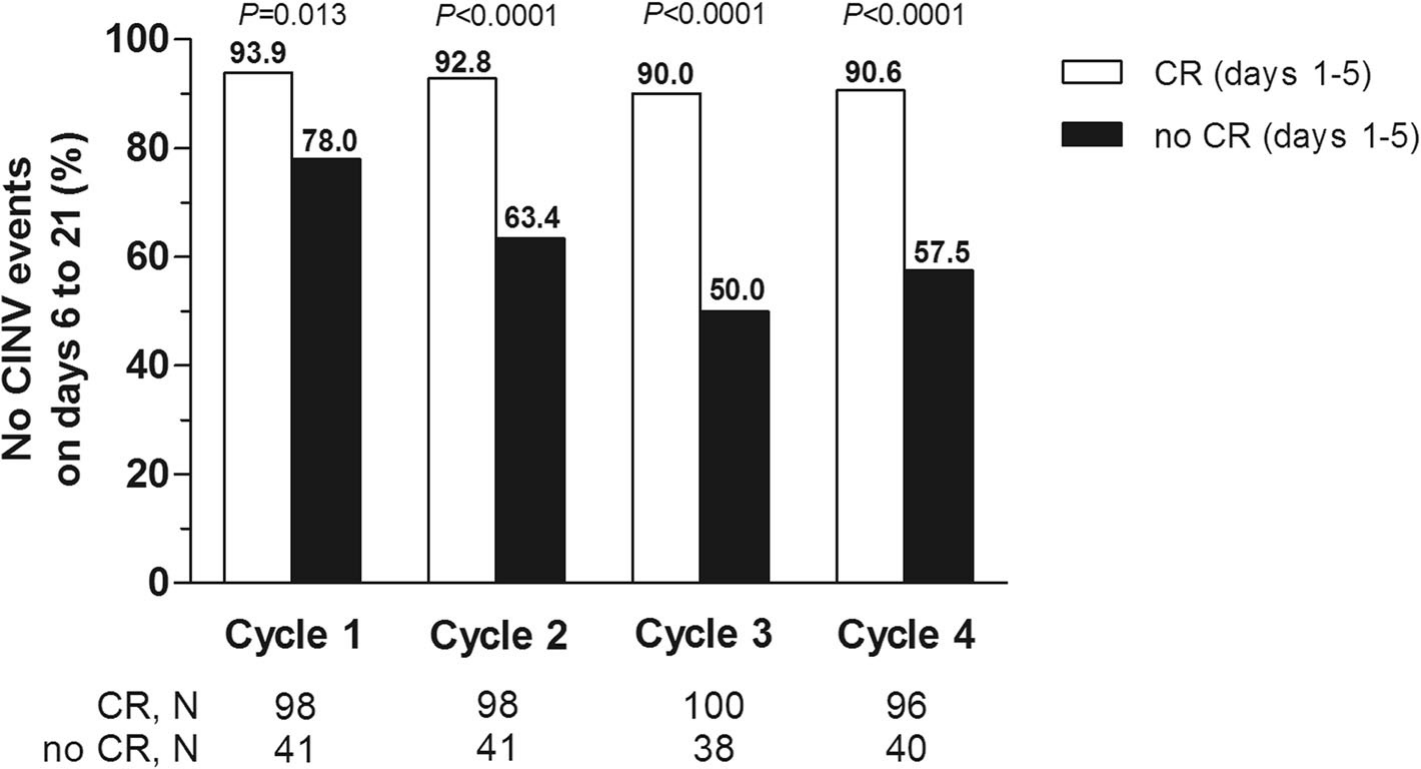
Proportion of patients without CINV events during the very late period by CR status seen in the overall period of each cycle. CINV, chemotherapy-induced nausea and vomiting; CR, complete response (no emesis, and no use of rescue medication). *P* values were calculated using the Fisher’s exact test (two-sided). CINV events were vomiting and/or moderate-to-severe nausea

The incidence of CINV events (i.e., vomiting and/or moderate-to-severe nausea) during the very late period increased over subsequent cycles (10.8% in cycle 1, 15.8% in cycle 2, 21% in cycle 3, and 19.1% in cycle 4). Overall, five patients experienced both nausea and vomiting, and further two patients had only vomiting in the very late period. In an exploratory analysis, the achievement of a CR during the overall study period was associated with a significantly better control of very late CINV over all chemotherapy cycles (Fig. 2).

A number of patient-related risk factors for CINV were included in a multivariable analysis to assess potential predictors for very late CINV among the patients with CR (Table 4). In this subgroup, the only predictor for increased likelihood of developing very late CINV events was pre-chemotherapy nausea. However, in cycles 1 and 2, no independent variable was found statistically significant. Conversely, in the third and fourth cycle, the occurrence of nausea (≥1 NRS) just before chemotherapy administration emerged as an independent predictor for very late CINV.

**Table 4:** Multivariable regression analyses on predictors of CINV in the very late period (days 6 to 21) in patients with a CR during the overall observation period

	Odds Ratioa	95% CI	***P***-value
Cycle 1 (*n* = 98)			
Motion sickness	1.50	0.21; 10.9	0.687
Pregnancy-related morning sickness	0.40	0.05; 3.00	0.376
Alcohol intake (every day vs. none)	Not estimable	Not estimable	Not estimable
Age (≥50 vs. < 50 years)	0.69	0.10; 4.90	0.707
Nausea over last 24 h (0–10 NRS)	0.74	0.53; 1.03	0.077
Anxiety over last 24 h (0–10 NRS)	0.88	0.66; 1.17	0.390
Cycle 2 (n = 98)			
Motion sickness	1.50	0.25; 9.07	0.660
Pregnancy-related morning sickness	0.51	0.10; 2.70	0.429
Alcohol intake (every day vs. none)	1.05	0.10; 11.6	0.968
Age (≥50 vs. < 50 years)	0.30	0.05; 1.86	0.197
Pre-chemotherapy nausea (0–10 NRS)	0.97	0.64; 1.47	0.870
Pre-chemotherapy anxiety (0–10 NRS)	0.92	0.65; 1.29	0.608
Cycle 3 (*n* = 100)			
Motion sickness	0.44	0.10; 1.97	0.281
Pregnancy-related morning sickness	0.67	0.16; 2.87	0.589
Alcohol intake (every day vs. none)	Not estimable	Not estimable	Not estimable
Age (≥50 vs. < 50 years)	2.16	0.47; 9.94	0.323
Pre-chemotherapy nausea (0–10 NRS)	0.65	0.50; 0.85	0.001
Pre-chemotherapy anxiety (0–10 NRS)	0.88	0.68; 1.15	0.354
Cycle 4 (*n* = 96)			
Motion sickness	0.11	0.01; 1.03	0.053
Pregnancy-related morning sickness	1.21	0.14; 10.6	0.862
Alcohol intake (every day vs. none)	0.77	0.04; 16.3	0.868
Age (≥50 vs. < 50 years)	0.11	0.01; 1.10	0.061
Pre-chemotherapy nausea (0–10 NRS)	0.50	0.34; 0.74	0.0006
Pre-chemotherapy anxiety (0–10 NRS)	1.16	0.78; 1.73	0.470

All patients received NEPA (netupitant/palonosetron) and dexamethasone on day 1 of each cycle; *CINV* chemotherapy-induced nausea and vomiting, *CR* complete response (no emesis and no use of rescue medication). *CI* confidence interval, *NRS* numeric rating scale (a score of 1 or greater indicating the occurrence of symptoms)

aOdds ratio lower than 1 indicates an increased likelihood of developing CINV events during the very late period

### Safety

A total of 146 patients were evaluable for safety in the study. Overall, NEPA was well tolerated over multiple cycles of AC chemotherapy without evidence for increasing adverse events (AEs) across cycles. In this study, the most common treatment-related AEs were fatigue (3.4%) and headache (2.1%). No patient discontinued treatment due to AEs, and no unexpected serious AEs occurred that could be attributed to the anti-emetic regimen across cycles.

## Discussion

The combination of AC is among standard chemotherapy regimens for the treatment of early breast cancer patients, and is now classified as HEC [4, 5]. In the present study, we selected women with early breast cancer receiving AC as adjuvant chemotherapy. It is important to underline that the majority of patients (55%) evaluated in this study was younger than 50 years of age, 32% had a history of motion sickness, and 38% of the patients had a history of pregnancy-related morning sickness. All these factors are known to be associated with a higher risk of developing CINV [14, 15]. Therefore, our patient population can be considered at particularly high risk for CINV.

NEPA, a combination of the new NK-1RA, netupitant and palonosetron, has been designed to improve guideline adherence by packaging guideline-recommended agents in a single oral fixed-dose. Since cancer patients usually receive multiple cycles of chemotherapy, this prospective, phase II study was conducted to evaluate whether the anti-emetic efficacy of NEPA would be maintained over subsequent cycles of AC. The prophylaxis with NEPA plus single-dose dexamethasone resulted in a CR rate of 71% during the overall study period in cycle 1, and was maintained through cycle 4. It is well known that the development of CINV in the first cycle of chemotherapy is a strong predictor of CINV in subsequent cycles [6]. In light of this, it is encouraging that the percentage of patients who experienced a CR in cycle 1 and who sustained a CR over cycles 2–4 was 53%. Interestingly, in a randomised pivotal trial of patients treated with AC, a triple regimen consisting of ondansetron, dexamethasone, and 3-day aprepitant resulted in a CR rate of 51% during the overall study period in cycle 1, while 35% of the patients sustained a CR over chemotherapy cycles 2–4 [13]. It is important to underline that the efficacy of NEPA plus single-dose dexamethasone observed in this study was generally in line with that observed in a recently published pivotal study evaluating the efficacy of the same anti-emetic regimen over multiple cycles of AC in a relatively homogeneous population of patients [16]. However, dropout rates, which can impact negatively on interpretation of results in multi-cycle studies [13], in the pivotal trial were relatively high, ranging from 12 to 24%, across cycles 2–4. In our study, 98% of the patients completed the planned 4 cycles of AC-containing chemotherapy, and the very low dropout rate reinforces the clinical relevance of the study findings. More recently, in a registration trial that assessed the safety of intravenous NEPA compared to oral NEPA, both in combination with single-dose dexamethasone, in breast cancer patients treated with AC, the proportion of patients receiving oral NEPA (*n* = 202) who achieved an overall CR ranged from 77 to 87% over cycles 1–4 [17]. However, only approximately 50% of patients completed all 4 cycles of treatment in this study.

In spite the advent of anti-emetics with novel mechanisms such as NK-1RAs, control of nausea still remains a clinical unmet need [18]. Since a very high-risk population for CINV was included in the current study, the rates of no significant nausea across cycles may be considered encouraging. The clinical benefit of NEPA against nausea over multiple cycles of AC is also supported by the previously mentioned pivotal trial where NEPA resulted in statistically significant superior rates of no significant nausea over palonosetron [16]. It is interesting to note that palonosetron plus dexamethasone was demonstrated to be superior to a first-generation 5-HT_3_RA plus dexamethasone for the control of nausea in the setting of AC [19].

An additional benefit of the anti-emetic prophylaxis with NEPA is to provide an opportunity to overcome barriers interfering with guideline adherence in clinical practice. More recently, an observational, prospective study showed that the prevention of CINV caused by AC is suboptimal in Italian clinical practice, with prophylaxis with aprepitant during the delayed period being administered to less than half of 246 patients with breast cancer [20]. This finding is consistent with data from a previous European observational study evaluating the use of a guideline-consistent prophylaxis in patients receiving emetogenic chemotherapy regimens [21]. Last but not least, a single-day prophylaxis with NEPA may help to overcome non-adherence to medications such as prescribed delayed anti-emetics that was identified as a prevalent issue among patients with breast cancer, particularly younger patients, and may have a cumulative effect on the occurrence and severity of CINV [22]. In light of this, NEPA could improve adherence to guidelines by minimising the overall pill burden for patients who fear that the action of swallowing itself would induce nausea and vomiting [23].

This prospective study provided insight into the importance of achieving CR in the overall period for the control of CINV events (i.e., vomiting and/or moderate-to-severe nausea) during the very late period (day 6 through 21) in each cycle of AC. In an exploratory analysis, no CINV events in the very late period occurred in 94% of the patients with a CR in cycle 1, with slightly lower incidence in later cycles of chemotherapy. In the subgroup of patients without a CR, significantly fewer patients were free from CINV events over the very late period in all chemotherapy cycles. These findings suggest that CR over the 5-day period of highest emetic risk after AC administration also plays an important part in the prevention of CINV over the very late period in each cycle. Among the patients with a CR, the predictive value of well-known risk factors for CINV was examined in a multivariable analysis for each cycle of AC. The only independent factor that negatively impacted the control of very late CINV was pre-chemotherapy nausea, with an effect observed from cycle 3 onwards. Pre-chemotherapy (anticipatory) nausea is a known risk factor for CINV, and a number of factors can place patients at higher risk of pre-chemotherapy nausea, including age, experiencing CINV in previous cycle, motion sickness, and female sex [12, 24]. Recently, a large, prospective observational study showed that pre-chemotherapy nausea is a predictor of CR in the acute, delayed, and overall periods alongside the use of guideline-consistent prophylaxis, younger age, and incomplete CINV response in an earlier cycle [6]. It should be noted that 21% of the patients in our study reported a score of 1 or greater for nausea on NRS just before receiving the first cycle of chemotherapy. Although this was also seen in recent, prospective, observational studies [12, 24], the clinical implications of this evidence still remain to be understood in CINV research.

## Conclusion

The results of this study indicate that the high anti-emetic efficacy seen with the NEPA regimen in the first cycle was maintained over multiple cycles of AC for breast cancer. Preliminary evidence suggests that the achievement of a CR in the overall period impacts also the risk for very late CINV in each cycle of AC. Therefore, CINV prevention over the 5-day period after chemotherapy administration remains a goal to improve control of symptoms for the whole duration of treatment cycle. As a single dose of NEPA and dexamethasone offers both effective and convenient guideline-consistent prophylaxis, future studies are warranted to determine the most feasible anti-emetic strategy to maximally prevent the nausea component of CINV over the entire risk period for each cycle of AC chemotherapy.

## Data Availability

The datasets used during the current study are available from the corresponding author on reasonable request.

## Abbreviations

AC: Anthracycline and cyclophosphamide
AE: Adverse event
CINV: Chemotherapy-induced nausea and vomiting
CR: Complete response
CTCAE: Common terminology criteria for adverse events
5-HT_3_RA: 5-hydroxytryptamine-3 receptor antagonist
HEC: Highly emetogenic chemotherapy
NK-1RA: Neurokinin-1 receptor antagonist
NRS: Numerical rating scale
TEAE: Treatment-emergent adverse event
